# High NRBP1 expression promotes proliferation and correlates with poor prognosis in bladder cancer

**DOI:** 10.7150/jca.32656

**Published:** 2019-07-10

**Authors:** Qi Wu, Xiaoqing Zhou, Peng Li, Wei Wang, Jun Wang, Mingyue Tan, Le Tao, Jianxin Qiu

**Affiliations:** 1 Department of Urology, Shanghai General Hospital of Nanjing Medical University, Shanghai 200080, China; 2 Department of Urology, The Sixth Affiliated Hospital of Wenzhou Medical University (The People's Hospital of Lishui), Zhejiang 323000, P. R. China; 3 Department of Urology, The Fourth Affiliated Hospital of Nantong University (Yancheng First People's Hospital), Jiangsu 224000, P. R. China; 4 Department of Urology, Shanghai General Hospital, Shanghai Jiao Tong University School of Medicine, Shanghai 200080, P. R. China

**Keywords:** Bladder cancer, NRBP1, Proliferation, Apoptosis, Survival.

## Abstract

Nuclear receptor binding protein 1 (NRBP1) is an evolutionarily highly conserved adaptor protein with multiple domains. Recently, its role in cancers has received increasing attention. To investigate whether NRBP1 is involved in the development of bladder cancer, we used tissue microarray and analyzed the association between the expression levels of NRBP1 and clinico-pathological features of 56 patients diagnosed with bladder cancer. Subsequently, NRBP1 was silenced using siRNA in bladder cancer cell lines T24 and 5637, and cell phenotype such as proliferation and apoptosis were observed. Further, in vivo tumor formation assay was performed. The expression of apoptosis markers was detected by Western blot. A significant positive correlation between increased NRBP1 expression and tumor stage, and lymph node metastasis was observed in 56 patients. High expression of NRBP1 was associated with poor prognosis and NRBP1 knockdown significantly inhibited cell proliferation and induced intrinsic apoptosis* in vitro*. Moreover, we also found that NRBP1 knockdown significantly suppress tumor growth in xenograft mouse model. Taken together, these data suggest that NRBP1 plays a critical role in the development of bladder cancer and may represent a potential target for bladder cancer treatment.

## 1. Introduction

Bladder cancer (BCa) is one of the most common malignant tumors affecting the urinary system, with increasing incidence rates. It ranks ninth in the incidence of malignant tumors worldwide, and fourth among cancers affecting the male gender 1. However, there is still insufficient understanding of the molecular mechanisms associated with the development of BCa. Although surgery and chemotherapy have improved the prognosis of BCa, the five-year survival rate of invasive BCa remains less than 50% 1. Therefore, there is an urgent need to discover new molecular markers for the diagnosis of bladder cancer.

Nuclear receptor binding protein 1 (NRBP1) is a highly conserved, ubiquitously expressed adaptor protein. The NRBP1 gene is located on human chromosome 2p23 and encodes 535aa protein. NRBP1 is considered to be a pseudokinase due to the lack of a key catalytic residue in the kinase core 2. Although NRBP1 lacks the ability to phosphorylate substrates, it forms a typical eukaryotic protein kinase fold and therefore, may retain other non-catalytic functions 3. In addition to the kinase-like domain, NRBP1 has a Src homology 2 (SH2) domain binding region, a binary nuclear localization signal, and three regions rich in proline, glutamate, serine, and threonine residues (PEST sequences) 2. NRBP1 binds to myeloid leukemia factor 1 (MLF1), JAB1 and activated RAC3, and is known to inhibit cell differentiation and JAB1-mediated AP1 activation and redistribution of the Golgi marker p58 4-6. Additionally, NRBP1 also interacts with Elongin BC and CUL5, the key molecules of ubiquitination, and is known to affect the cellular protein concentration of TSC22d2 and SALL4, suggesting its putative role in the turnover of these proteins 7.

Interestingly, NRBP1 is known to have different role in the tumor progression in different tumors. *Nrbp1* is considered to be an intestinal tumor suppressor gene as per the intestinal tumorigenic phenotype of the *Nrbp1* conditional knockout mice 7. NRBP1 is known to inhibit the progression of breast cancer, lung adenocarcinoma and certain types of lymphomas 8, 9, but has the opposite effect in prostate cancer 10, 11. Therefore, the specific mechanism of action of NRBP1 is still poorly understood in different tumor subtypes. The role of NRBP1 in the progression of bladder cancer has not yet been elucidated. Using the survival analysis of the TCGA database, we found that the high expression level of NRBP1 is associated with poor prognosis in bladder cancer. Therefore, we hypothesized that NRBP1 might function as a tumor promoting gene in bladder cancer.

## 2. Materials and Methods

### 2.1. Clinical sample collection

20 fresh primary BCa specimens and corresponding non-tumorous tissues (at least 5cm away from the edge of the tumor) were collected after surgical resection at Lishui People's Hospital (Zhejiang, China) between November 2016 and May 2018. Fresh tissues were collected and immediately stored at -80°C after resection for subsequent RNA extraction. 46 paraffin-embedded BCa specimens and 10 cancer and paracancerous paired samples from January 2007 to October 2014 were obtained from Shanghai General Hospital (Shanghai, China) for tissue microarray (TMA) analysis. No patients recruited in the study received other treatments. Two pathologists examined and confirmed the scoring of the cancer tissues and matched normal tissues. Tumor stage and grade assessments were made according to the World Health Organization (WHO) 1973 standard and the American Joint Committee on Cancer (AJCC) 2002 TNM system. All patients signed the informed consent. Protocols for use of human surgical samples were approved by the Medical Ethics Committee of Shanghai General Hospital and Lishui People's Hospital.

### 2.2. Follow-up

Physical examination and laboratory investigation of 56 patients was carried out every 3-6 months for the first 5 years and every 12 months thereafter during the follow-up period. All the patients were followed up until the end of the study (December 30, 2014) or eventual death. The overall survival was calculated from the date of diagnosis to the date of death, the last known survival or study deadline. The median follow-up time in this study was 35.7 months (range: 3-82).

### 2.3. TMA and immunohistochemistry (IHC)

56 BCa tissues were selected for TMA construction, including 10 matched cancer and paracancerous tissue specimens and 46 cancer specimens. After hematoxylin and eosin staining, the samples were screened for optimal tumor content for the construction of tissue microarrays. A core was taken from a central sample of each paraffin-embedded bladder tumor lesion and its adjacent non-tumor samples using a punched core having a maximum dimension of 2.0 mm.

Immunohistochemistry was carried out using a two-step method as described previously 12. After heat-induced antigen retrieval, TMA was incubated with NRBP1 primary antibody (1:100, Abnova) for 30 minutes at room temperature, followed by incubation with a matched secondary antibody for 30 minutes. The sections were observed under a microscope and developed in 3, 3'-diaminobenzidine solution (Sigma) and counterstained with hematoxylin (Sigma). The immunoreactivity of the NRBP1 protein in TMA was independently reviewed and scored by two experienced pathologists, who were blinded to clinical data and patient data. A total of 10 representative microscopic fields (or the tumor cells in the entire core if number of tumor cells are limited) were counted, averaged, and summarized. The expression NRBP1 was then semi-quantified using the immunoreactivity score (IRS) system. The staining score ranged from 0 to 4, corresponding to the percentage of immunoreactive tumor cells (0, 1, 2, 3, and 4 corresponding to 0%, 1-5%, 6-25%, 26-75%, and 76-100% respectively). The staining intensity scores were as follows: negative (score = 0), weak (score = 1), medium (score = 2) or strong (score = 3). A score of 0 to 12 was calculated by multiplying the staining degree score by the intensity score.

### 2.4. Tissue RNA isolation and quantitative real-time PCR (qRT-PCR)

Total RNA was extracted from the tissues with Spin Column Animal Total RNA Purification Kit (Sangon, Shanghai, China) following manufacturer's specifications. Reverse transcription was performed using the PrimeScript RT Master Mix (Takara, Shiga, Japan) followed by qRT-PCR using PowerUp SYBR Green Master Mix (Thermo Fisher Scientific, Waltham, MA, USA) and QuantStudio 7 Flex Real-Time PCR system (Thermo Fisher Scientifc). β-actin was used as an internal control. The primers of NRBP1 and β-actin were purchased from Sangon Inc. Primers for NRBP1 are 5′-GGACTCATCAAGATTGGCTCTG-3′ (forward) and 5′-TCTTCTGCTCTTCTCGACAAGT-3′ (reverse). Primers for β-actin are 5′-CATGTACGTTGCTATCCAGGC-3′ (forward) and 5′-CTCCTTAATGTCACGCACGAT-3′ (reverse).

### 2.5. Cell culture and reagents

Human bladder cancer T24 and 5637 cell lines were purchased from the Chinese Academy of Science (Shanghai, China) and cultured in RPMI 1640 (Gibco, Gaithersburg, MD, USA) supplemented with 10% fetal bovine serum (FBS; Biological Industries, M.P. Ashrat, Israel). Cells were cultured at 37 ℃ in a humidified environment with 5% CO_2_.

### 2.6. NRBP1 siRNA and Transfection

Two pairs of short interfering RNA (siRNA) targeting NRBP1 sequence (siNRBP1-1: CCTTGAAGATGTCAGGAAT; siNRBP1-2: GTCGAGAAGAGCAGAAGAA) were synthesized by Genepharm Inc. (Shanghai, China). SiRNA targeting NRBP1 or scrambled siRNA (negative control, siCONT) were transfected using Lipofectamine RNAiMAX transfection reagent (Invitrogen, Carlsbad, CA, USA) to knock down endogenous NRBP1. Total protein was extracted from cells to analyze NRBP1 silencing efficiency.

### 2.7. Protein Extraction and Western Blot

Cellular protein was extracted from the cells transfected with or without siNRBP1 using RIPA lysis buffer (ThermoFisher Scientific, Waltham, MA, USA). After protein quantification with Pierce BCA Protein Assay Kit (ThermoFisher Scientific), the protein lysate was separated by SDS-PAGE, transferred to a 0.22μm PVDF membrane (Millipore, Billerica, MA), and used for the expression analysis of NRBP1 (Abnova, Taipei, Taiwan), β-actin (Sangon), Cleaved-caspase 3, 8, 9, Cleaved-PARP, Cytochrome c, BAX, and BCL2 (Cell Signaling Technology, Danvers, MA). The primary antibody was detected after incubation with horseradish peroxidase-conjugated secondary antibody. Bound antibodies were visualized using an enhanced chemiluminescence system (Merck Millipore, Darmstadt, Germany).

### 2.8. Cell proliferation assays

After transfection with control or targeting siRNA for 16h. At 24, 48, 72, 96, 120h in 6cm dishes, T24 and 5637 cells were transferred to 96-well plates at a density of 2000 cells per well in triplicates. Cell proliferation was assessed using the Cell Counting Kit-8 kit (Dojindo Laboratories, Kyushu Island, Japan) according to the manufacturer's instructions. The results are reflected in the form of the absorbance optical density at 450 nm using a microplate reader (Synergy H1; BioTek, VT, USA).

According to the manual of Cell-Light EdU Apollo 567 In Vitro Imaging Kit (Ribobio, Guangzhou, China), 50 μM EdU labeling medium was added to the cell culture and cultured for 2 hours under normal culture conditions. Thereafter, the cells were fixed in 4% paraformaldehyde for 30 minutes, followed by incubation with glycine for 5 minutes. After washing with PBS, the cells were stained with an anti-EdU working solution for 30 minutes at room temperature, washed in PBS containing 0.5% Triton X-100, and further incubated with Hoechst 33342 dye for 30 minutes at room temperature. The specimens were then observed under a fluorescence microscope (Axiouret S100, Carl Zeiss, Germany). The percentage of EdU positive cells was calculated as average from five randomly selected fields in three wells.

### 2.9. Flow cytometry assay

Apoptosis assay was performed by flow cytometry. 48h after transfection with siNRBP1 or siCONT, the cells were stained with Annexin V-FITC and propidium iodide (BD Biosciences, San Diego, CA, USA), according to manufacturer's instructions to detect early and late apoptosis. The extent of cell apoptosis, indicated as the percentage of apoptotic cells, was measured using Accuri C6 flow cytometer (BD). All experiments were performed in triplicates.

### 2.10. Lentiviral vector construction and animal experiments

More efficient siRNA (siNRBP1-2) was selected for construction of a lentiviral vector (Genechem, Shanghai, China) and the viral transfection procedure was performed according to the instructions provided by the company. Knockdown efficiency was detected by qPCR and Western blot. Stably expressing LV-shNRBP1/T24 cells and their vector control cells (LV-shCONT/T24) were used to produce the xenograft models. Stable cells (5×10^6^) in 50 µl PBS mixed with Matrigel (4:1) were subcutaneously injected into 6-week-old male nude mice (nu/nu) mice (SLAC, Shanghai, China) (n = 5). The tumor volume was measured about every 3 days. After 6 weeks, the mice were sacrificed, and the xenografts were removed and subjected to immunohistochemical detection of NRBP1, Ki-67 and Cleaved-caspase3.

### 2.11. Statistical analyses

The results were analyzed using IBM SPSS Statistical Software for Windows (version 22.0, SPSS Inc., Chicago, IL, USA) and are presented as mean ± standard error of the mean of three independent experiments performed in triplicate. The correlation between NRBP1 expression level and clinic-pathological parameters was evaluated by Pearson χ^2^ test. Kaplan-Meier method was used to plot the survival curves and they were compared with the log-rank test. Univariate and multivariate analysis was performed by applying Cox proportional hazards test. Two-tailed Student's t-test was used to assess the statistical significance of differences in groups. *P*<0.05 was considered as statistically significant.

## 3. Results

### 3.1. NRBP1 expression was up-regulated in bladder cancer tissues

**Figure 1:**
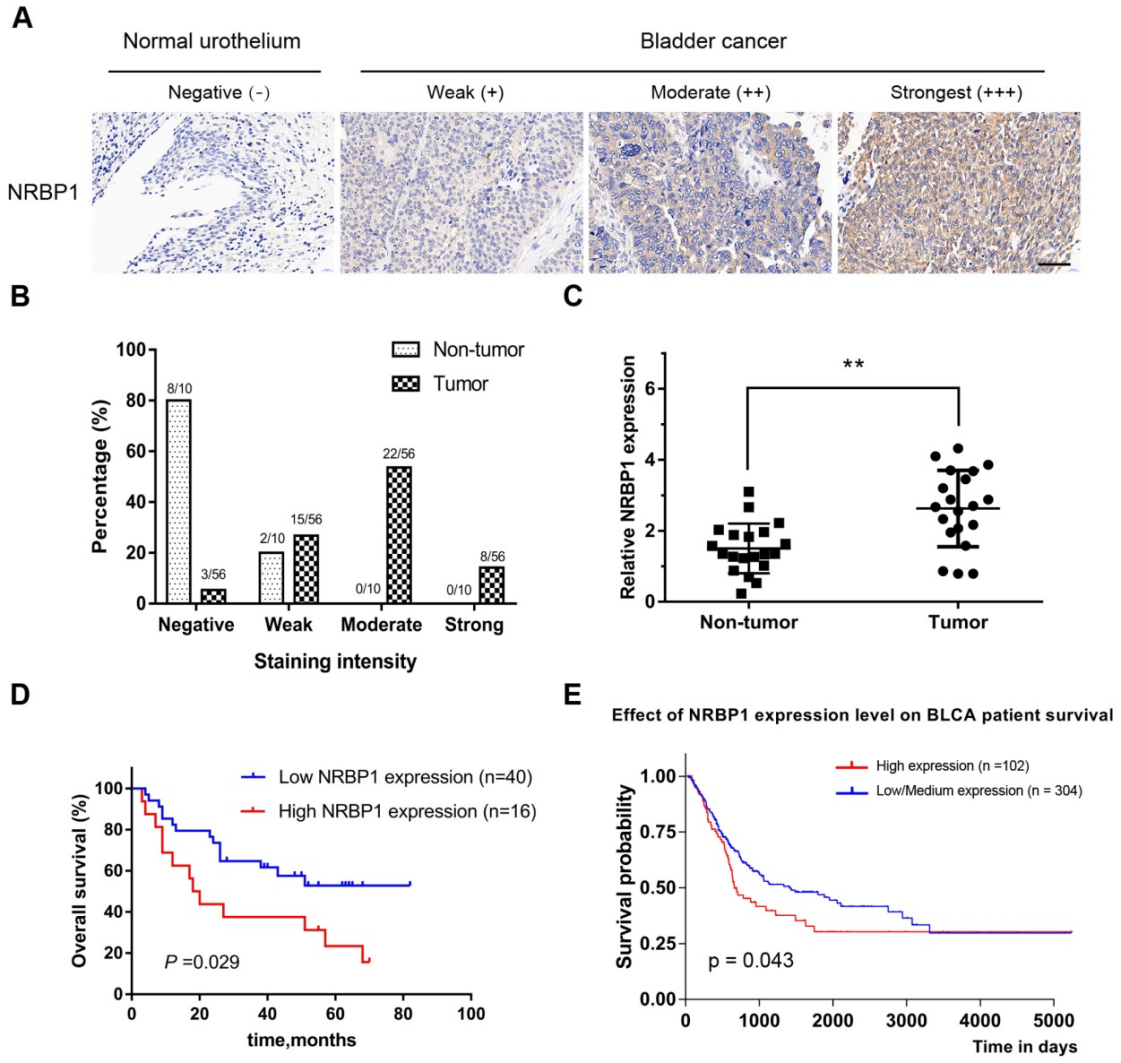
The expression levels of NRBP1 are up-regulated in BCa tissues. (A) Immunohistochemical staining for the Expression of NRBP1 in normal bladder urothelium and BCa tissues (brown). TMA sections were counterstained with hematoxylin. Scale bar: 100μm. (B) The stained TMA sections were scored into four groups according to the staining intensity, and NRBP1 protein levels were higher in BCa tissues. (C) The mRNA expression of NRBP1 by qRT-PCR in 20 paired BCa tissues and adjacent non-tumor tissues. (D) Kaplan-Meier analysis for increased NRBP1 expression and its association with poor overall survival in BCa patients.* P* = 0.029, log-rank test. (E) Data from TCGA indicating the association of high expression of NRBP1 with poor overall survival.

Immunohistochemical analysis of TMA showed the positive staining intensity for NRBP1 in BCa tissues as significantly stronger than the staining intensity in the corresponding adjacent tissues. Among 56 cases of BCa, moderate and strong staining accounted for 53.6% (30/56), while only negative (8/10) or weak staining (2/10) was observed in the 10 corresponding adjacent cancer tissues. (Moderate/strong staining group vs Weak/negative staining group, *P* =0.0017, Chi-square test), (Fig. 1A, B). These results suggest that NRBP1 protein levels are relatively higher in BCa tissues compared with normal bladder urothelium. We also found that NRBP1 was mainly localized in the cytoplasm, but not in the nucleus.

To further clarify the expression level of NRBP1 mRNA in BCa and paracancerous tissues, we performed qRT-PCR on 20 pairs of cancer and adjacent tissues. NRBP1 mRNA expression was higher in BCa tissues than the transcript levels in the adjacent tissues (Fig. 1C). Taken together, the results indicate an upregulation of NRBP1 in BCa tissues.

### 3.2. High expression of NRBP1 is associated with poor prognosis of bladder cancer

Next, the association between NRBP1 expression and the clinico-pathological data was performed (Table 1) and indicated that high NRBP1 expression was correlated with the tumor stage and lymph node metastasis (*P* = 0.03 and *P* = 0.002, respectively), but not significantly associated with other parameters such as age (*P* = 0.33), gender (*P* = 0.91), tumor diameter (*P* = 0.84), or tumor grade (*P* = 0.12). Subsequently, we analyzed the association between NRBP1 expression and patient survival. 56 patients were followed up for a mean of 35.7 months and Kaplan-Meier analysis coupled with log-rank test showed that the patients with high NRBP1 expression had poor prognosis than those with low expression of NRBP1(*P* = 0.029), indicating that NRBP1 expression was significantly associated with overall survival in BCa patients (Fig. 1D).

Finally, we analyzed the correlation between the expression levels of NRBP1 in patients with bladder urothelial carcinoma (BLCA)and the overall survival using the Cancer Genome Atlas (TCGA) database, and found that low-medium expression of NRBP1 was associated with better prognosis (Fig. 1E). In the univariate analysis, NRBP1 expression (*P* < 0.001), tumor grade (*P* < 0.001), tumor stage (*P* < 0.001), and lymph node metastasis (*P* < 0.001) were the statistically significant predictors for overall survival (Table 2). Multivariate Cox proportional regression analysis revealed that NRBP1 expression (*P* = 0.014), age (*P* = 0.021), tumor grade (*P* = 0.011), and lymph node metastasis (*P* = 0.006) are independent prognostic factors for the overall survival of BCa patients (Table 2).

### 3.3. Knockdown of NRBP1 inhibited proliferation of bladder cancer cell lines and promoted apoptosis *in vitro*

**Figure 2:**
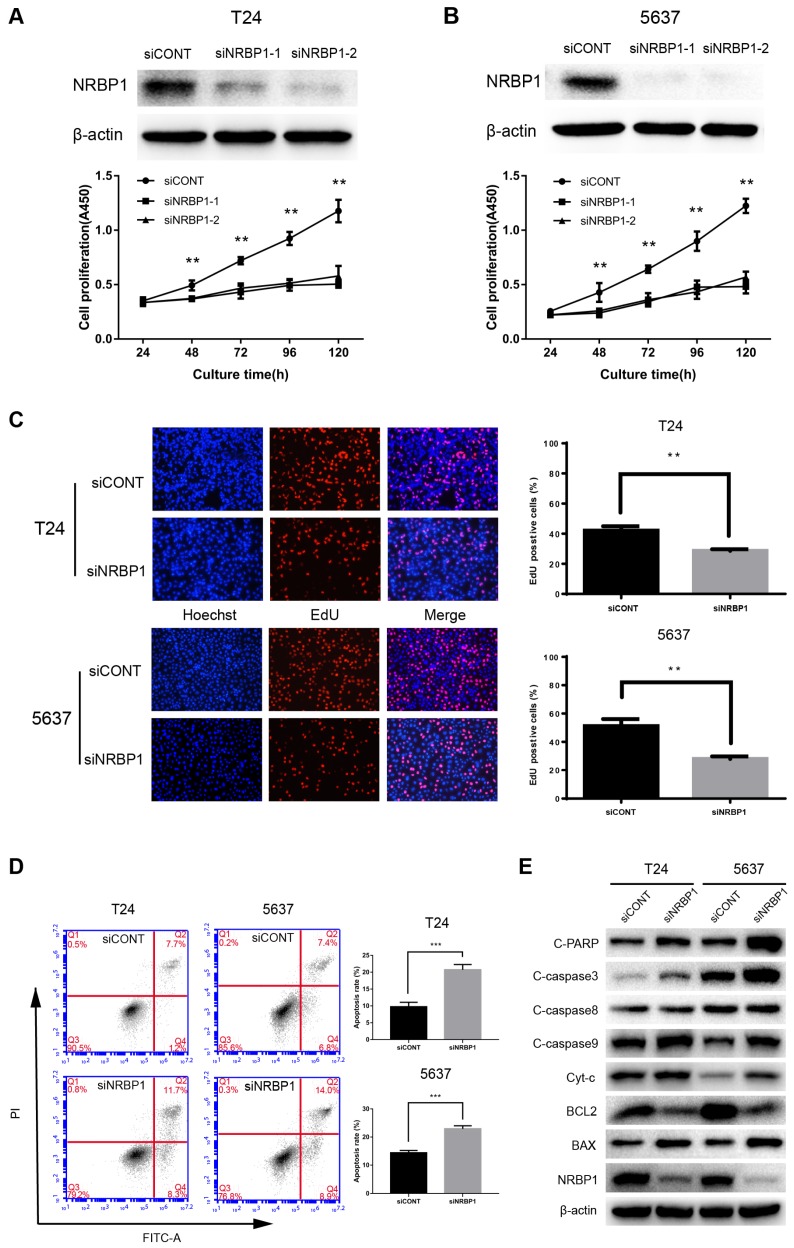
Knockdown of NRBP1 inhibits the proliferation and induces apoptosis in bladder cancer cells. (A, B) NRBP1 protein levels in T24 and 5637 cells transfected with two siRNAs 48 hours post-transfection (top). The CCK-8 cell proliferation assay in siCONT or siNRBP1 group of T24 and 5637 cells at indicated time points. (C) Representative images of EdU staining in T24 and 5637 cells labeled with EdU (red) and Hoechst 33342 (blue) (Magnification 200×). The percentage of EdU positive cells were quantified, and data are expressed as mean ± SE (n = 6). (D) Apoptotic cell measurement by flow cytometry 48 h after transfection. The cell populations of Annexin V-FITC and PI were used to assess apoptotic events. (** *P* < 0.01 and ***P < 0.001). (E) Examination of the protein expression levels of intrinsic apoptosis markers in T24 and 5637 cells after NRBP1 knockdown. Beta-actin was used as a loading control.

To further illustrate the biological significance of NRBP1 in bladder cancer, we performed gene silencing of NRBP1 by siRNA. We analyzed the mRNA and protein levels to select the optimal concentration of siRNA to knock down NRBP1 mRNA. These concentrations were used for subsequent functional studies. As can be seen in Figures 2A-B, after 48-120 hours of transfection with siRNA, the siNRBP1 group showed a significantly slower proliferation rate than the control group. Consistent with the results of proliferation assay, the control group had a higher proportion of EdU-positive cells than the siNRBP1 group (Fig. 2C). Moreover, flow cytometry analysis revealed that NRBP1 knockdown remarkably induced cell apoptosis in T24 and 5637 cells (Fig. 2D).

### 3.4. Inhibition of NRBP1 enhances expression of markers associated with the intrinsic apoptotic pathway

We explored the potential role of NRBP1 in apoptosis of bladder cancer cells by Western blot analysis. NRBP1 knockdown significantly increased the protein levels of cleaved caspase-3, caspase-9 and PARP, while the level of cleaved capase-8 remained unchanged, suggesting activation of the intrinsic apoptotic pathway. The key markers of the intrinsic apoptotic pathway (BCL-2 and BAX) were down-regulated and up-regulated, respectively, accompanied with an increase in cytoplasmic cytochrome *c* (Cyt-c) (Fig. 2E).

### 3.5. NRBP1 knockdown suppressed tumor growth of bladder cancer cells* in vivo*

**Figure 3:**
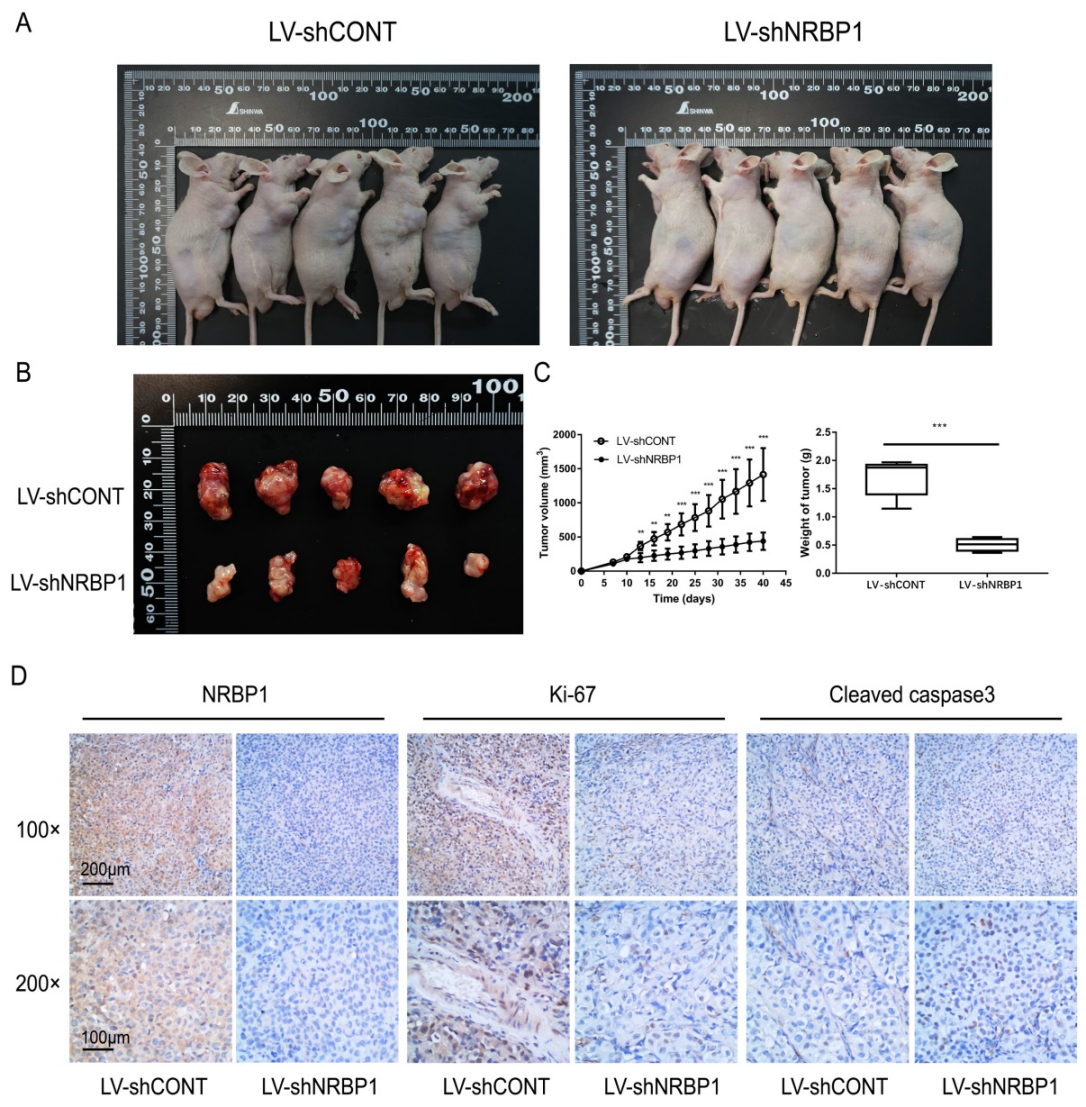
Knockdown of NRBP1 suppressed bladder tumor growth *in vivo*. A: Photographs of tumor bearing nude mice inoculated with T24/LV-shCONT and T24/LV-shNRBP1 cells (n = 5). B: Tumors tissues isolated from the above two groups of nude mice (n = 5). C: Tumor volumes and weights of LV-shCONT and LV-shNRBP1 groups (n = 5). D: Representative images of NRBP1, Ki-67 and cleaved-caspase3 in tumor tissues from LV-shCONT and LV-shNRBP1 mice detected by IHC staining. (^**^P < 0.01, ^***^P < 0.001).

To investigate whether NRBP1 affects tumor cell growth in vivo, we first constructed a lentiviral vector of shNRBP1 and its control shRNA (shCONT). After infection of T24 cells, knockdown efficiency was verified by qPCR and Western blot. After 6 weeks of observation, we concluded that consistent with *in vitro* experiments, the tumor growth was slower in the LV-shNRBP1 group compared with the LV-shCONT group (Fig. 3A-C). Subsequent immunohistochemistry experiments also confirmed that the LV-shNRBP1 group had lower expression of Ki-67 compared with the control group, accompanied by higher expression of Cleaved-caspase3 (Fig. 3D).

## 4. Discussion

Our study demonstrated for the first time, that high NRBP1 expression in BCa patients was associated with poor prognosis and adverse histopathological parameters. To confirm the hypothesis that NRBP1 promotes BCa progression, we silenced NRBP1 in the bladder cancer cell lines with siRNA and observed cell proliferation and apoptosis. Transfection of two bladder cancer cell lines (T24 and 5637) with siNRBP1 significantly inhibited the proliferation and promoted intrinsic apoptosis *in vitro* and* in vivo*, demonstrating the potential anti-apoptotic role of NRBP1 in promoting proliferation of bladder cancer cells.

Apoptosis is regulated by two major pathways: the intrinsic mitochondrial pathway and the extrinsic death receptor pathway 13. Cysteine protease centrally regulates cell apoptosis. Caspase-3 acts as an effector caspase, activated by caspase-9 in the intrinsic pathway and caspase-8 in the extrinsic pathway 14. The intrinsic pathway is regulated by the BCL-2 protein family, and releases cytochrome *c* from the mitochondria into the cytoplasm, leading to caspase activation 15. Our results showed that down-regulation of NRBP1 induced the intrinsic apoptotic pathway in bladder cancer cells. These results are however inconsistent with the recent findings on NRBP1 in colorectal cancer 9. This phenotypic difference may be related to differences in protein expression and signaling pathways in different cancer subtypes.

NRBP1 is considered to be a tumor suppressor gene in several other NRBP1 cancer-related studies, such as breast cancer and lung cancer 7, 8 but is known to promote prostate cancer cell proliferation 10. Since the research on NRBP1 and cancer has just begun, the exact regulatory mechanism is still largely unknown. However, it is undeniable that NRBP1 plays an important role in the regulation of cell growth due to its high degree of conservation and the characteristics of multiple protein domains. For example, *Madm,* the human NRBP1 homolog in* Drosophila*, interacts with Bunched A (human long TSC22DF homolog of *Drosophila*) to synergistically regulate the growth of *Drosophila* eyes and wings 16. In addition, there is a hypothesis that NRBP1 may act as a scaffolding protein for Elongin BC E3 ubiquitin ligase to promote ubiquitination of specific substrates 7. Ubiquitin ligases often recognize a variety of substrates and mediate substrate degradation or participate in the regulation of signaling pathways 17. Therefore, it is not surprising that NRBP1 has different mechanisms in different cancers.

Furthermore, NRBP1 is primarily expressed in the cytoplasm, which is consistent with most previous studies 4, 6, 10. Since NRBP1 has putative nuclear localization signal (NLS) and nuclear export signal (NES) sequences, it is thought to shuttle between the cytoplasm and the nucleus 2. As a result, NRBP1 may also have the function of binding to specific ligands for their intracellular transport, which may also be closely related to cell proliferation.

In conclusion, our data demonstrates that NRBP1 expression is upregulated in BCa tissues and NRBP1 knockdown inhibits bladder cancer cell proliferation and promotes cell apoptosis* in vitro* and in vivo. High expression of NRBP1 was associated with poor prognosis in BCa patients. Our findings suggest that NRBP1 is essential in the development of bladder cancer and its role as a novel diagnostic marker in BCa needs to be further investigated.

## Figures and Tables

**Table 1:** Clinicopathological correlation of NRBP1 expression in bladder cancer

			NRBP1 expression		
Parameter		No. of cases	Low	High	*P*-value
Age, years					0.33
<65		19	12	7	
≥65		37	28	9	
Gender					0.91
Male		46	33	13	
Female		10	7	3	
Tumor diameter (cm)					0.84
<5		42	30	12	
≥5		14	10	4	
Tumor grade					0.13
Grade2		30	24	6	
Grade3		26	16	10	
Tumor stage					0.03
T1-2		27	23	4	
T3-4		29	17	12	
LN metastasis					0.002
N0		41	34	7	
N1		15	6	9	

Statistical significance (*P* <0.05) is shown in bold

**Table 2:** Univariate and multivariate analysis of the overall survival using Cox proportional hazard model

Variable	Univariate analysis		Multivariate analysis	
	*P*-value		Hazard ratio (95% CI)	*P*-value
NRBP1 expression (low vs. high)	**<0.001**		3.625 (1.304-10.075)	**0.014**
Age, years (<65 vs. ≥ 65)	0.603		1.067 (1.010-1.127)	**0.021**
Gender (male vs. female)	0.283		0.755 (0.270-2.111)	0.592
Tumor diameter (<5cm vs. ≥5cm)	0.969		1.241 (0.415-3.709)	0.7
Tumor grade (grade2 vs. grade3)	**<0.001**		5.255 (1.460-18.907)	**0.011**
Tumor stage (T1-2 vs. T3-4)	**<0.001**		3.936 (0.894-17.320)	0.07
LN metastasis (N0 vs. N1)	**<0.001**		9.462 (1.885-47.483)	**0.006**
